# Combined Analysis of Transcriptome and Metabolome Provides Insights in Response Mechanism under Heat Stress in Avocado (*Persea americana* Mill.)

**DOI:** 10.3390/ijms251910312

**Published:** 2024-09-25

**Authors:** Xinyi Zheng, Qing Zhu, Yi Liu, Junxiang Chen, Lingxia Wang, Yu Xiu, Haoyue Zheng, Shanzhi Lin, Peng Ling, Minqiang Tang

**Affiliations:** 1 Key Laboratory of Genetics and Germplasm Innovation of Tropical Special Forest Trees and Ornamental Plants (Ministry of Education), Hainan Key Laboratory for Biology of Tropical Ornamental Plant Germplasm, Collaborative Innovation Center, School of Tropical Agriculture and Forestry, Hainan University, Haikou 570228, China; 23220954000032@hainanu.edu.cn (X.Z.); 22210907000001@hainanu.edu.cn (Q.Z.); 22220954000016@hainanu.edu.cn (Y.L.); cjunxiang20l@gmail.com (J.C.); wlxhnu2000@163.com (L.W.); 21220954000049@hainanu.edu.cn (H.Z.); 2 College of Biological Sciences and Biotechnology, National Engineering Laboratory for Tree Breeding, Key Laboratory of Genetics and Breeding in Forest Trees and Ornamental Plants, Ministry of Education, Tree and Ornamental Plant Breeding and Biotechnology Laboratory of National Forestry and Grassland Administration, Beijing Forestry University, Beijing 100083, Chinaszlin@bjfu.edu.cn (S.L.)

**Keywords:** avocado, transcriptome, metabolomic analysis, heat stress, differentially expressed metabolites

## Abstract

Plants generate a range of physiological and molecular responses to sustain their growth and development when suffering heat stress. Avocado is a type of tropical fruit tree with high economic value. Most avocado cultivars delete, wither, or even die when exposed to heat stress for a long time, which seriously restricts the introduction and cultivation of avocados. In this study, samples of a heat-intolerant variety (‘*Hass*’) were treated under heat stress, and the transcriptomics and metabolomics were analyzed, with the expectation of providing information on the variety improvement and domestication of avocados. The differentially expressed genes identified using transcriptome analysis mainly involved metabolic pathways such as plant hormone signal transduction, plant–pathogen interaction, and protein processing in the endoplasmic reticulum. Combined transcriptome and metabolome analysis indicated that the down-regulation of *Hass.g03.10206* and *Hass.g03.10205* in heat shock-like proteins may result in the reduced Trehalose and Sinapoyl aldehyde content. Metabolomics analysis results indicated that the decrease in Trehalose and Sinapoyl aldehyde content may be an important factor for heat intolerance. These results provide important clues for understanding the physiological mechanisms of adaptation to heat stress in avocados.

## 1. Introduction

The 2023 IPCC Sixth Assessment Report emphasizes the increasingly significant impacts of climate change and warns that global temperatures are likely to rise to dangerous levels, posing serious threats to ecosystems and human livelihoods (https://www.ipcc.ch/, accessed on 18 September 2024). With the increasing trend of global warming, heat stress poses a severe challenge to crop production and seriously threatens the normal growth and development of crops, resulting in various direct and irreversible damages to plant growth and development [1]. High temperatures seriously impair the function of plants in regulating adversity, and they will eventually cause heat damage and even mortality in fruit trees. Heat stress has gradually become one of the adverse factors affecting the high yield, high efficiency, and sustainable development of the fruit tree industry [2].

Avocado (*Persea americana* Mill.) is known for its rich nutrients and is widely cultivated in tropical and subtropical regions as an important economic crop [3,4]. With the planting area and yield of avocados increasing, it has become a popular fruit variety with important economic significance. It is urgent to cultivate high-quality avocado varieties and improve the yield and quality of avocados. According to the differences in the ecological conditions of origin, avocados are mainly divided into Guatemalan, Mexican, and West Indian families [5]. ‘*Hass*’, derived from Mexican varieties, is the largest commercial avocado in the world, accounting for about 65% of the world’s avocado consumption [6]. ‘*Hass*’ is relatively sensitive to heat; when it is exposed to high-temperature conditions, the growth of the seedling might exhibit thermodormancy or even death, which seriously restricts its planting area and production [7,8].

Under moderate and high-temperature stress, drastic damages occur in membrane thermostability as an indicator of heat injury and oxidative stress [9,10]. In order to help plants cope with high temperatures, it is important to improve the thermal stability of lipid membranes. The stability of lipid membranes is affected by the saturation or unsaturation of membrane fatty acids, and high levels of saturated fatty acids can enhance the heat resistance of membranes [11,12]. The fatty acid desaturase (FAD) family in plants plays a role in adapting to high stress and regulating membrane fluidity [8]. Furthermore, elevated temperatures can influence the interactions between lipids and proteins, activate signaling transduction pathways, and alter gene expression as well as transcript accumulation. Ca^2+^ and transcription factors regulate cellular signal transduction under high stress. Heat shock transcription factor (HSF) is a conserved protein in all eukaryotes studied so far and is the most important transcription factor [9]. HSF can activate heat shock response and induce the production of heat shock protein (HSP). The antioxidant system plays a key role in high stress, protecting plants from oxidative damage through antioxidants and enzymes. Plant hormones and osmoregulants also regulate the ability of plants to cope with high temperatures and affect the role of plant hormone pathways and regulators [13,14,15]. These comprehensive regulatory mechanisms play an important role in plants’ response to stress in high-temperature environments. These studies have provided a basis for understanding the molecular mechanisms of plant response to high stress. Therefore, taking the ‘*Hass*’ variety as the research object, combined with omics technology, can help to fill the knowledge gap regarding how high temperature affects avocado development. At present, research on avocado mainly focuses on seedling breeding techniques, new variety selection, cultivation techniques, and tissue culture techniques [16,17,18]. Therefore, it is necessary to study the changes in genes and metabolism of avocado under high-temperature stress.

Based on the above research background and research foundations, the purpose of this study is to (a) analyze the phenotypic and physiological changes of avocado under heat stress; (b) identify differentially expressed genes (DEGs) in response to heat stress and differentially expressed metabolites (DEMs) and their key metabolic pathways; (c) explore the molecular mechanisms of heat stress tolerance in avocado. The purpose of this study was to explore the key genes, metabolites, and related pathways of avocado under heat stress, so as to provide a scientific basis for genetic improvement in avocado and the study of heat stress in other plants.

## 2. Results

### 2.1. Phenotype of Leaves under Heat Stress

**Figure 1:**
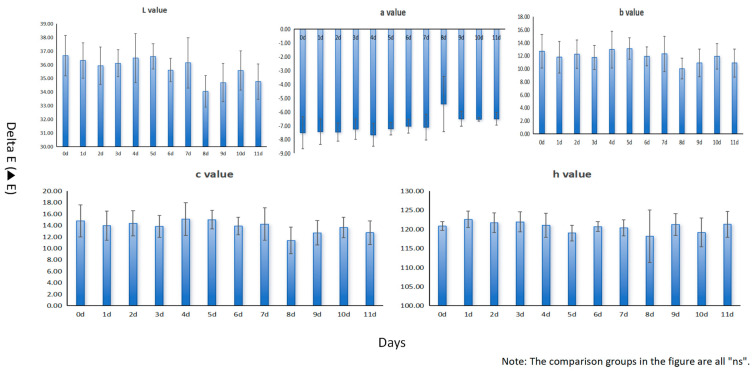
Color changes of leaves at different heat stress times. The horizontal axis represents different heat stress times from 0 to 11 days, and the vertical axis represents the color parameters of L value, a value, b value, c value, and h value, respectively. The L value represents the brightness of the sample, with higher values indicating a brighter color. The a value represents the red-green color degree of the sample, with positive values indicating a red bias and negative values indicating a green bias. The b value represents the yellow-blue color degree of the sample, with positive values indicating a yellow bias and negative values indicating a blue bias. The c value represents the chroma of the sample, with higher values indicating a more saturated color. The h value represents the color phase of the sample, with numerical values indicating color angles.

Three repeated samples were measured for L value (brightness), a value (red-green color degree), b value (yellow-blue color degree), c value (chroma), and h value (color phase) for 11 days by using a colorimeter, revealing the color changes of leaves under heat stress at different times. According to the chart (Figure 1 and Table S1), under heat stress, the color parameter values of the leaves show slight fluctuations, indicating that their color has been affected. In the early stage of heat stress, the changes in L value, b value, and h value are relatively slow, and, as time goes on, the range of changes gradually increases; meanwhile, the changes in a value and c value are more significant in the early stage of heat stress, and as time passes, the range of changes gradually decreases. However, on the whole, the phenotypic changes in leaves are not significant. Therefore, it is necessary to comprehensively analyze other physiological and biochemical parameters in addition to the phenotype. When plants experience heat stress, the changes in transcription levels will be significant in a short time, and a long period of data collection will produce a large amount of data, which will lead to complexity in data processing and analysis. Therefore, the main transcription responses can be observed within the first two days.

### 2.2. Transcriptome Sequencing and Analysis

With a total of 51.55 Gb clean reads, nine samples had equal bases and no AT/GC separation. The identification accuracy was above 99.9%, Q30 sequences were higher than 96.31%, and the alignment efficiency of clean sequences in nine samples ranged from 93.65% to 94.17% (Table S2). A total of 3228 genes with expression changes were detected by transcriptome sequencing before and after two days of heat treatment. A total of 2655 differentially expressed genes were detected after 1d heat stress, of which 1409 were up-regulated genes and 1246 were down-regulated genes. A total of 573 differentially expressed genes were detected between the 1d and 2d samples, of which 311 were up-regulated genes and 262 were down-regulated genes (Tables S3–S5).

**Figure 2:**
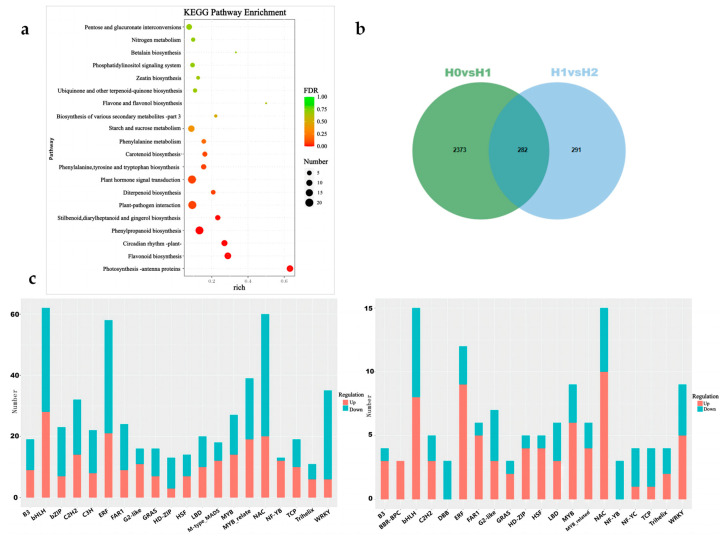
Results of gene expression patterns and KEGG enrichment analyses: (**a**) KEGG enrichment analysis plot for H0 vs. H1 comparison group. According to the KEGG enrichment results, the degree of enrichment is measured by the Rich factor, FDR value, and the number of genes enriched on this pathway. Among them, the Rich factor refers to the ratio of the number of enriched differentially expressed genes in the pathway to the number of annotated differentially expressed genes. The larger the Rich factor, the greater the degree of enrichment becomes. The general range of FDR values is 0–1, and the closer it is to zero, the more significant the enrichment; (**b**) Venn diagram of differentially expressed genes between H0 vs. H1 and H1 vs. H2 groups; (**c**) Bar graph of differential transcription factors. The left graph represents the H0 vs. H1 group and the right graph represents the H1 vs. H2 group. The horizontal axis represents different transcription factor families, and the vertical axis represents the number of genes belonging to each transcription factor family.

Enrichment analysis of differentially expressed genes was performed by referring to the KEGG pathway phylogenomic information database. As can be seen from Figure 2a, 2655 differentially expressed genes were mainly distributed in plant hormone signal transduction, plant–pathogen interaction, protein processing in the endoplasmic reticulum, pentose and glucuronate interconversions, and photosynthesis. In the metabolic routes of antenna proteins (pigment-binding proteins in the photosynthetic system that capture light energy and optimize the efficiency of photosynthesis) [19,20], phenylpropanoid biosynthesis, and starch and sucrose metabolism. The 573 differentially expressed genes detected in the 1d and 2d comparison groups were mainly collected in the metabolic routes of plant–pathogen interaction, phenylpropanoid biosynthesis, tryptophan metabolism, starch and sucrose metabolism, and protein processing in the endoplasmic reticulum. KEGG results showed that the heat resistance of avocado under heat stress was mainly due to the decrease in metabolic pathways such as plant hormone signal transduction, plant–pathogen interaction, and protein processing in the endoplasmic reticulum, thus shortening its continuous heat resistance time.

A Venn diagram analysis of differentially expressed genes in response to heat stress in avocado at different times (Figure 2b) revealed that 282 common differentially expressed genes were detected in the H0 vs. H1 and H1 vs. H2. There were 2373 and 291 specific differentially expressed genes detected in the comparisons of H0 vs. H1 and H1 vs. H2, respectively. With the extension of heat stress, the number of differentially expressed genes involved in the response to high stress decreased accordingly.

The differentially expressed genes predicted to be transcription factors were counted, and the number of differentially expressed transcription factors belonging to various families in the comparison group was shown in the bar chart (Figure 2c). We identified 36 different types of common transcription factors. Among them are the main transcription factor families, including bHLH, NAC, ERF, MYB, and WRKY. Through the analysis of transcriptome data, it was found that the number of differentially expressed transcription factors decreased after 2 days of heat stress compared to 1 day. This phenomenon indicates that avocado may have limited sustained heat tolerance.

### 2.3. Metabolomic Analysis of Heat Stress

According to Tables S6–S8, the analysis of metabolomics showed that a total of 623 metabolites were detected in this experiment, there were 33 differentially expressed metabolites after the treatment with heat stress for 1 day, and 21 of them were up-regulated. Among the comparison groups after 1d and 2d of heat stress treatment, 48 differentially expressed metabolites were detected, and 18 of them were up-regulated. With the prolongation of high-stress time, the differentially expressed metabolites increased.

**Figure 3:**
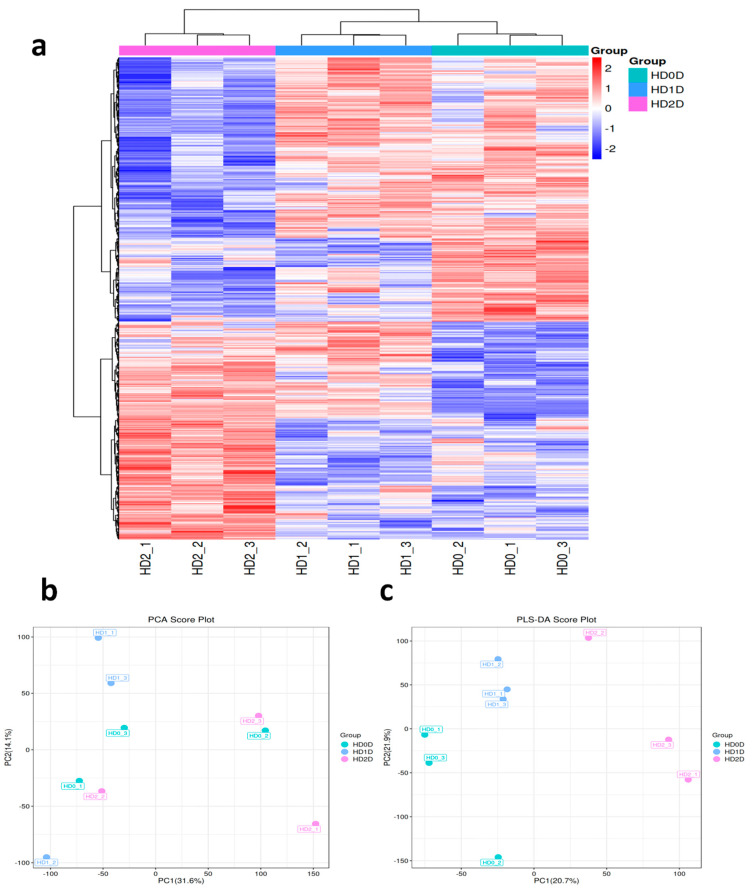
Quality control of metabolomics data and content of metabolites: (**a**) Heatmap of the dem cluster analysis results. In the matrix, columns represent samples and rows represent metabolites. The clustering tree on the left displays the clustering of different metabolites, while the clustering tree at the top represents the clustering of samples. The gradient colors indicate the magnitude of the quantitative values; the deeper the red, the higher the expression level, while the deeper the blue, the lower the expression level. Metabolite names are not displayed when the number of metabolites exceeds 150; (**b**) DEM principal component analysis; (**c**) DEM analysis based on PLS-DA score. The x-axis (PC1) represents the scores of the first principal component, and the y-axis (PC2) represents the scores of the second principal component. Each point symbolizes a sample, the shaded area denotes the 95% confidence interval, and the colors indicate different groups.

To better understand the change patterns of metabolites under heat stress in avocado, the untargeted metabolomics study was performed. First, the metabolite content was standardized to construct a hierarchical clustering heat map (Figure 3a). Principal component analysis (PCA) and partial least squares discriminant analysis (PLS-DA) were subsequently performed on the clustering information of the three treatment groups. The results showed that there was a significant separation between the H0, H1, and H2 treatments (Figure 3b,c), which indicated that the metabolites in the samples changed significantly after different heat stress treatments.

**Figure 4:**
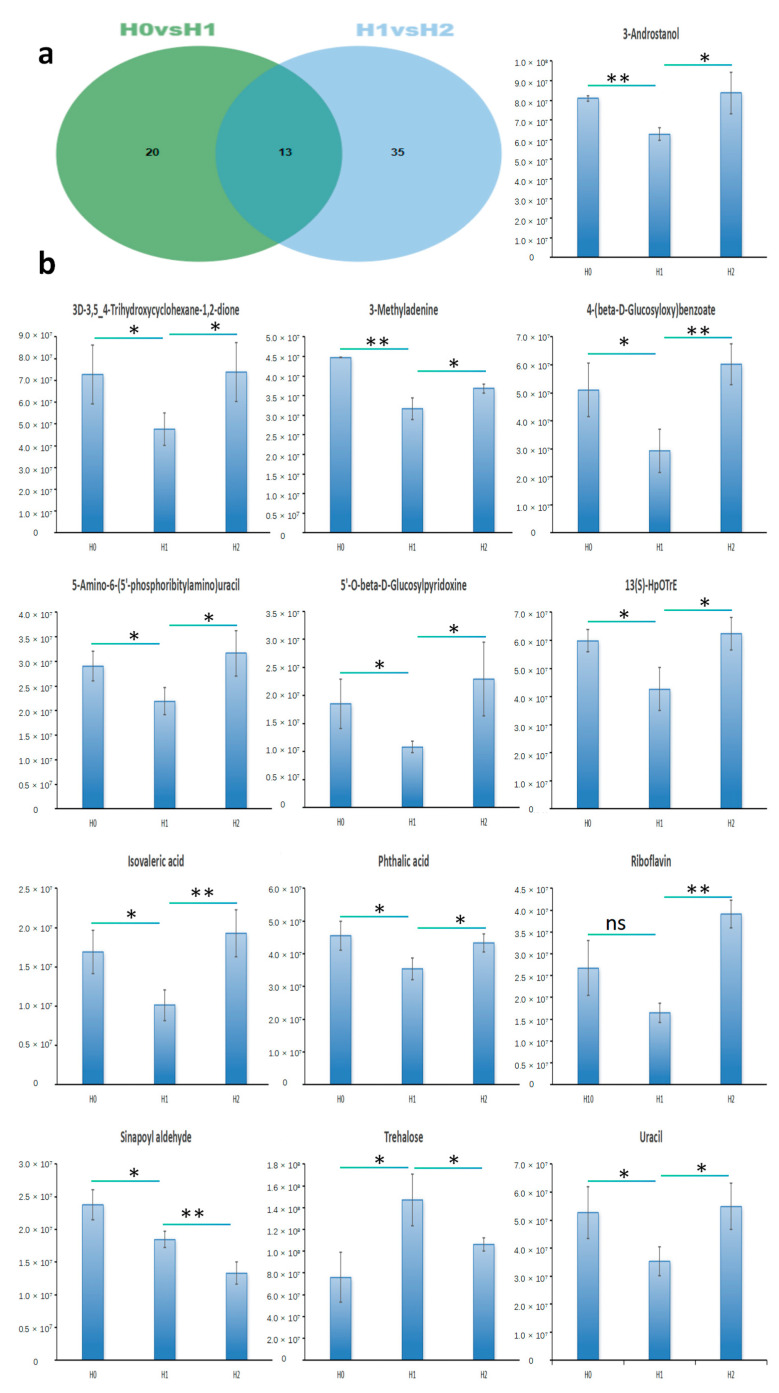
Common metabolites: (**a**) Venn diagram of high-temperature metabolites in H0 vs. H1 and H1 vs. H2 groups; (**b**) changes in the content of common metabolites of H0 vs. H1 and H1 vs. H2 comparison groups over time under heat stress. The abscissa represents the change time of metabolite content from 0 to 2d, and the ordinate represents the relative content of metabolites. “*” indicates statistical significance, *p* ≤ 0.05. “**” indicates stronger statistical significance, *p* ≤ 0.01. While “ns” denotes non-significance, *p* > 0.05.

According to the effects of the time of heat stress on differentially expressed metabolites (Figure 4a and Table S9): 13 common metabolites were detected in both H0 vs. H1 and H1 vs. H2. They were 13(S)-HpOTrE, 3-Androstanol, 3-Methyladenine, 3D-3,5_4-Trihydroxycyclohexane-1,2-dione, 4-(beta-D-Glucosyloxy)benzoate, 5′-O-beta-D-Glucosylpyridoxine, 5-Amino-6-(5′-phosphoribitylamino)uracil, Isovaleric acid, Phthalic acid, Riboflavin, Sinapoyl aldehyde, Trehalose, and Uracil, respectively. According to the results of metabolomics research, changes in metabolite content are as follows (Figure 4b): In addition to Trehalose and Sinapoyl aldehyde, the levels of the other 11 metabolites decreased on the first day of heat stress but showed an increase by the second day. This may be attributed to the prolonged duration of heat stress, which encourages the gradual development of heat tolerance in these metabolites. In the comparison of H0 vs. H1, the content of Trehalose was significantly increased, whereas it significantly decreased in the comparison of H1 vs. H2, The content of Sinapoyl aldehyde gradually decreases as the duration of heat stress increases. Revealing that the decrease in Trehalose and Sinapoyl aldehyde might be one of the main contributing factors of avocado heat intolerance. At the same time, through the annotation of the KEGG pathway database, Trehalose is enriched in two metabolic pathways of ABC transporters and starch and sucrose metabolism, while Sinapoyl aldehyde is enriched in phenylpropanoid biosynthesis.

### 2.4. Analysis of Trehalose and Sinapoyl Aldehyde Metabolite Content Change and Gene Regulation in Response to Heat Stress

**Figure 5:**
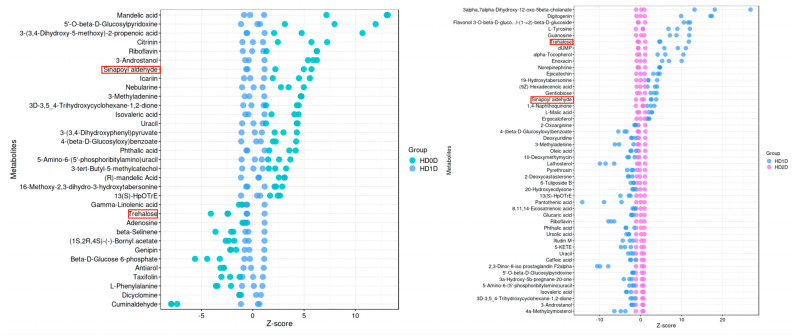
Z-score plot of the H0 vs. H1 and H1 vs. H2 comparison groups. The key metabolites, Trehalose and Sinapoyl Aldehyde, are highlighted in red boxes. The coordinates are then converted Z-score values of the relative content of the metabolite in the sample, the ordinate is the metabolite name, and the colors of the points represent different groups. The closer to the **right side** indicates the higher relative abundance of the current metabolite in this sample, and the closer to the **left side** indicates the lower abundance of the current metabolite.

Relevant research has shown that there was a significant correlation between Trehalose and the heat stress response of plants [21,22,23,24]. Based on the existing data, we cannot directly confirm that there is a significant correlation between Sinapoyl aldehyde and the heat stress response of plants. However, according to study [25], the accumulation of this metabolite may be part of the plant’s strategy to maintain photosynthetic activity and combat oxidative stress. Combining the results of transcription and metabolomics, we inferred that the decreased content of Trehalose and Sinapoyl aldehyde might be one of the main reasons for the heat intolerance of avocado varieties. As shown in Figure 5, the content of Trehalose increased significantly one day after the heat stress, but it decreased significantly on the second day after the stress; the content of Sinapoyl aldehyde decreased as the stress time extended.

**Figure 6:**
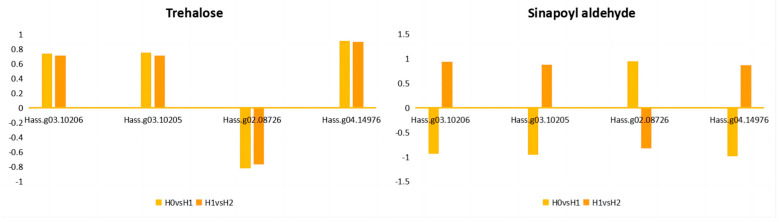
The correlation between Trehalose and Sinapoyl aldehyde and their common genes. The abscissa represents different genes, and the ordinate represents the correlation coefficient of different genes. A positive value represents a positive correlation, and a negative value represents a negative correlation. The greater the absolute value, the stronger the correlation.

Trehalose was positively correlated with most genes at two time points, such as *Hass.g03.10206*, *Hass.g03.10205*, and *Hass.g04.14976*. The correlation coefficients between expression and Trehalose content were 0.74, 0.75, and 0.91 (H0 vs. H1), and 0.71, 0.71, and 0.90 (H1 vs. H2), respectively. However, Trehalose was negatively correlated with the *Hass.g02.08726* gene at both time points, with correlation coefficients of −0.82 and −0.77, respectively. Sinapoyl aldehyde showed a more dynamic change. The correlation coefficients with *Hass.g03.10206*, *Hass.g03.10205*, and *Hass.g04.14976* genes were negative at H0 vs. H1, which were −0.94, −0.96, and −0.99, respectively, but turned positive at H1 vs. H2, which were 0.94, 0.88, and 0.87, respectively. On the contrary, the correlation with the *Hass.g02.08726* gene was positive (0.95) at the H0 vs. H1 time point, but negative (−0.83) at the H1 vs. H2 time point (Figure 6).

According to Table 1 and Table S10, in the H0 vs. H1 control group, the expression of DEGs associated with *Hass.g03.10206* and *Hass.g03.10205* genes in heat shock-like protein is up-regulated, while in the H1 vs. H2 control group, the expression of these two DEGs is down-regulated. Additionally, in the H0 vs. H1 group, the expression of *Hass.g02.08726* gene in geraniol 8-hydroxylase-like protein is down-regulated, while the expression of *Hass.g04.14976* gene in putative aquaporin PIP2-8 is up-regulated. In the H1 vs. H2 control group, the expression of *Hass.g02.08726* and *Hass.g04.14976* genes shows the opposite trend. This indicated that the down-regulation of *Hass.g03.10206* and *Hass.g03.10205* genes in heat shock-like protein may reduce Trehalose and Sinapoyl aldehyde content, and the changes in gene expression of geraniol 8-hydroxylase-like protein and putative aquaporinPIP2-8 also affect Trehalose and Sinapoyl aldehyde content, thereby affecting the heat tolerance performance of avocado under heat stress.

## 3. Discussion

### 3.1. The Physiological Response and Transport Response of Avocado to Heat Stress

Observations made on avocado, after subjecting them to varying durations of heat stress, revealed a noteworthy stability in their physiological responses. Specifically, there were no marked changes during the observation period, and the avocado leaves retained their verdant hue even after enduring 11 days of heat stress. This stability could be attributed to the insufficient intensity or duration of the heat stress, necessitating a comprehensive evaluation encompassing other pertinent physiological and biochemical indicators.

Metabolome analysis studies found a continuous increase in other metabolites under heat stress except that Trehalose and Sinapoyl aldehyde decreased in the H1 vs. H2 contrast group. These metabolites are mainly 5′-O-beta-D-Glucosylpyridoxine, 3-Androstanol, and 3D-3,5_4-Trihydroxycyclohexane-1,2-dione, which stand out as they play pivotal roles in sustaining plant vitality under prolonged heat stress conditions [26]. However, the reduction in the content of Trehalose and Sinapoyl aldehyde destroyed the metabolism of plants.

### 3.2. The Key Role and Mechanism of Trehalose and Sinapoyl Aldehyde in Heat Stress Response in Avocado

Under heat stress conditions, the physiological and biochemical mechanisms within plants undergo alterations of varying magnitudes. Extensive research indicates that the diminution in chlorophyll content resulting from heat stress serves as a deterrent to photosynthesis, subsequently impeding plant growth [27].

To counter high temperatures, plants increase soluble sugar concentrations to maintain cellular osmolarity and adjust cell membrane fluidity, thereby minimizing heat stress damage [28,29]. Soluble sugar accumulation in the cytoplasm is essential for protein hydration and preventing protoplasm dehydration. It balances osmotic potential between the cytoplasm and vacuoles, stabilizing membrane fluctuations and boosting the plant’s resilience to high temperatures [30]. Consequently, we hypothesized that the reduction in soluble sugars might affect the resistance of avocado samples to heat stress. Trehalose is a non-reducing disaccharide with the molecular formula C_12_H_22_O_11_H_2_O, a relative molecular mass of 378.33, white crystal, stable chemical properties, and is non-toxic and pollution-free. It is a storage sugar in plants, bacteria, and fungi, and is essential for stress metabolism, protecting cellular components from damage in adverse conditions like heat, drought, freezing, and high osmotic pressure [31,32]. Specifically, the concentration of Trehalose increased in the H0 vs. H1 comparison group, while the Trehalose concentration decreased in the H1 vs. H2 comparison group, which suggests that Trehalose plays a protective role under the initial heat stress; however, its concentration decreases with the stress duration, probably due to the inadaptation of plants to persistent heat stress. Through KEGG pathway database annotation, it was found that Trehalose was mainly enriched in two metabolic pathways: ABC transporters and starch and sucrose metabolism. These pathways play an important role in the plant’s response to heat stress. ABC transporter protein, as a key transmembrane protein, can participate in the electron transport and carbon fixation process of the photosynthetic system under heat stress, thus ensuring that photosynthesis can proceed normally under high-temperature conditions [33]. In addition, further studies suggest that ABC transporters may indirectly affect the efficiency of photosynthesis by regulating the activities of related enzymes, providing strong support for plant survival and adaptation in extreme environments [34]. Regarding, starch and sucrose metabolism, the dynamic balance of sucrose and starch in plants is essential for maintaining normal physiological functions under heat stress. For example, heat stress leads to accelerated starch decomposition and increased sucrose content, which may be a plant attempt to increase the osmolarity pressure of cells by increasing soluble sugars to reduce the damage caused by high temperature [35].

Sinapoyl aldehyde is a compound involved in secondary metabolism in plants, which is usually converted to corresponding aldehydes by binding with coenzyme A and then reduced to alcohols [36]. Through KEGG pathway database annotation, it was found that sinapoyl aldehyde was mainly enriched in phenylpropanoid biosynthesis. This pathway is mainly based on the aromatic amino acids phenylalanine (in most plants) or tyrosine (in some monocotyledons) as precursors, through a series of enzymatic reactions to produce a variety of secondary metabolites, including flavonoids, lignans, phenolic acids, resveratrol, and coumarin [37]. These compounds play an important role in plants, and, for example, as an important part of the cell wall, a protective agent against high light and ultraviolet radiation, a defensive substance against herbivores and pathogens, and anthocyanins that mediate the interaction between plants and pollinators [38,39].

Heat stress may also affect other metabolic pathways, such as Pantothenate and CoA biosynthesis, beta-alanine metabolism, phenylalanine, tyrosine and tryptophan biosynthesis, and Pyrimidine metabolism, which also play an important role in the plant response to environmental stress. The metabolic response of avocados under heat stress mainly included changes in Trehalose and Sinapoyl aldehyde concentration, the enrichment of ABC transporters, starch and sucrose metabolic and phenylpropanoid biosynthesis pathways, and the modulation of other related metabolic pathways. Together, these metabolic changes constitute the integrated mechanism of response to heat stress.

### 3.3. Transcriptome Response of Avocado to Heat Stress

Plants activate the expression of specific genes in response to high-temperature stress, thereby changing their morphological, physiological, and biochemical properties [40,41,42]. We performed transcriptome analysis on leaf samples under different high-temperature treatments and detected a large number of DEGs under H0 vs. H1 (Table S3), which indicates that avocado has a positive response to high-temperature stress on the first day, which can stimulate its defense mechanism. KEGG enrichment results showed that the DEGs associated with avocado sample resistance to high temperature were mainly enriched in plant hormone signal transduction, plant–pathogen interaction, and protein processing in the endoplasmic reticulum (Figure 2a). The results of the analysis in this study differ from other studies, which may be due to differences in species [43,44]. Plant hormone signal transduction is one of the important mechanisms for plants to respond to environmental stress. For example, abscisic acid (ABA) and salicylic acid (SA) are two major phytohormones that play a key role in the plant response to abiotic stresses such as drought and salt stress. Under heat stress, plants enhance their thermotolerance by regulating the levels of these hormones [45,46,47]. Thus, when these signal transduction pathways are reduced, plants may be unable to effectively modulate hormone levels that affect their thermotolerance. Plant–pathogen interaction involves complex immune response processes, which include the involvement of multiple signaling molecules and proteins. This interaction may be disturbed at heat stress, which can alter the mode of interaction between the pathogen and the host to affect the plant’s immune response [48]. Protein processing in the endoplasmic reticulum is essential for maintaining the proper folding and function of intracellular proteins. Under heat stress, the correct folding of the protein may be affected, leading to protein accumulation and cellular dysfunction. In addition, heat stress may increase the production of reactive oxygen species (ROS), further impairing protein structure and function [49]. The reduction in these metabolic pathways may lead to the inability of plants to effectively respond to heat stress, thereby affecting their growth, development, and yield. Therefore, in-depth studies of the specific mechanisms of the action of these metabolic pathways could facilitate the development of new strategies to improve plant heat tolerance to address the challenges posed by global climate change.

Transcription factors play important roles in the plant’s response to stress environments. These transcription factors might play important roles in heat stress response, regulating plant fitness and stress resistance through the regulation of gene expression. For example, TCP transcription factors are involved in the regulation of plant growth and development and leaf morphology and also play an important role under stress [50,51,52]. The bHLH transcription factors play a key regulatory role in stress response, flowering bloom, and light signaling in plants [53,54]. In this study, a total of 3228 transcription factors were detected, among which TCP, bHLH, and NAC were the most abundant families (Table S11), and we speculate that they play an important role in the resistance of avocado samples to heat stress.

### 3.4. Analysis of Gene Regulation of Avocado in Response to Heat Stress

The specific mechanisms of up- to down-regulated gene expression in plants under heat stress involve multiple levels, including the regulation of the transcriptional level, the activation of signaling pathways, and the response of specific gene families. Heat stress triggers a variety of physiological and molecular responses in plants. To enhance their tolerance to heat stress, plants produce heat shock proteins (HSPs) that assist in maintaining proper protein folding within cells, safeguarding them from heat-induced damage [55,56]. For example, heat stress prompts the expression of genes like HSP, ribosomal protein L12, and elongation factor EF-Tu, along with those linked to photosynthesis in *Porphyra haitanensis* [41]. Despite the up-regulation of certain genes, many are down-regulated under high-temperature stress, including those involved in nucleic acid, protein, and carbohydrate synthesis in *Porphyra haitanensis* [41]. In terms of signaling, heat stress activates multiple signaling pathways that are involved in regulating the plant response to high temperature through different mechanisms. For example, studies have shown that plants activate gene expression networks associated with heat sensation, heat stress signaling, and heat regulation under heat stress to adapt to elevated environmental temperatures [57]. The study of genes related to female ear development in sweet corn found that 244 genes were down-regulated under heat stress. The down-regulated expression of these genes may reflect the effect of heat stress on plant growth and development processes [58]. This down-regulation of gene expression may be a strategy adopted by plants to reduce energy expenditure and protect limited energy resources.

The differential expression of the ABA 8′-hydroxylase gene in various plants and its biological importance is mainly reflected in its impact on plant growth and development and environmental adaptability. ABA is a key hormone in plants, which is involved in regulating a variety of physiological processes of plants, including growth and development and coping with stress [59,60]. The regulation of ABA level is jointly controlled by its synthesis and degradation pathway, while the degradation of ABA is mainly regulated by 8′-hydroxylase, which belongs to the P450 enzyme. There were significant differences in the expression patterns of the ABA 8′-hydroxylase gene among different plant species. For example, in legumes, pvcyp707a1, pvcyp707a2, and pvcyp707a3 genes are regulated under water stress and heavy water treatment, indicating that they play a key role in plants’ response to environmental changes [61]. In soybean, the inhibition of the ABA 8′-hydroxylase gene can increase ABA accumulation and improve plant tolerance to salt stress [62]. This highlights the importance of the ABA 8′-hydroxylase gene in plant adaptation to abiotic stress. Studies on the expression of the putative aquaporin PIP2 gene have shown that this gene family plays an important role in plant reproductive organs, roots, leaves, and other tissues. Taking tobacco as an example, the expressions of PIP1 and PIP2 aquaporins were significantly different during anther and stigma development, and ntpip2; 1 showed high water channel activity [63]. In addition, the increased expression of oepip2.1 in the olive dwarf genotype was associated with hydraulic conductivity [64]. In wheat straw, the overexpression of pvpip2; 9 increased biomass yield, protein content, and drought resistance [65]. The study also found that, in alfalfa, the mspip2;2 gene improved plant salt tolerance by regulating the antioxidant defense system and ion balance under salt stress [66]. These results support the important role of PIP2 aquaporins in plant adaptation to environmental stress. To conclude, despite the current absence of direct investigative studies focusing on the 8-hydroxylase-like protein and the hypothetical aquaporin PIP2-8, our functional research into the relevant protein genes sheds new light on their significance. Specifically, it becomes evident that PIP2-8 occupies a pivotal position in regulating plant water homeostasis and stress responses. Furthermore, the varied expression of the ABA 8′-hydroxylase gene has profound effects on plant physiological and biochemical pathways by precisely modulating ABA levels, thereby exerting a critical influence on how plants adapt and respond to dynamic environmental conditions. At the same time, heat stress commonly impacts key proteins associated with photosynthesis, such as Rubisco and Photosystem II. In this study, we observed that the expression level of Rubisco was significantly higher in the H0VSH1 group compared to the H1VSH2 group, indicating that the plant’s ability to adapt to prolonged high-temperature conditions is impaired. Similarly, the abundance of Photosystem II proteins was also relatively high in the H0VSH1 group, whereas it decreased markedly in the H1VSH2 group, which may directly affect the plant’s photosynthetic efficiency and growth. Thus, the changes in the expression of Rubisco and Photosystem II could be important factors contributing to the heat intolerance observed in *Hass* under high-temperature conditions.

Therefore, through previous studies, combined with the transcriptomic and metabonomic results of this study, we found that the reduction in Trehalose and Sinapoyl aldehyde in the two control groups was one of the main components of avocado heat intolerance. The analysis of common genes of the common metabolite Trehalose and Sinapoyl aldehyde showed that all common genes changed in the two control groups. Specifically, in the H0 vs. H1 control group, the differential expression genes (DEGs) of *Hass.g03.10206* and *Hass.g03.10205* genes in the heat shock-like protein were up-regulated, while, in the H1 vs. H2 control group, they were down-regulated. This suggests that the down-regulation of *Hass.g03.10206* and *Hass.g03.10205* genes in heat shock-like protein may lead to a decrease in Trehalose and Sinapoyl aldehyde content, while the up-regulation may help avocado adapt to high-temperature environments. The specific mechanism of avocado gene expression changes under high-temperature stress involves complex signal transduction pathways and diverse transcriptional regulation mechanisms. These mechanisms include not only gene expression changes that directly respond to high-temperature stress, but also strategies to adapt to high-temperature environments by regulating basic metabolism and biosynthesis.

## 4. Materials and Methods

### 4.1. Plant Materials and Treatments

The plant materials selected in this experiment are approximately 70 cm tall saplings of the ‘*Hass*’ variety. During the seedling stage, fertilize once with mature organic compost. Water the plants 2–3 times a week, and, during periods of high temperatures, use shade nets to provide some protection from direct sunlight. Afterwards, seedlings were placed in an incubator at 27 °C, 16 h light, 8 h dark, and 75% humidity. After seven days, the temperature was adjusted to 35 °C. The precision colorimeter (NR60CP, 3nh Technology Co., Ltd., Shenzhen, China) was used to measure the color change of the fully unfolded and healthy leaves (about third to last leaves from the top) with three repeats. Leaves with three repetitions were collected at different time points (0 days, 1 day, and 2 days). All samples were stored in liquid nitrogen for the sequencing of transcriptome and metabolome.

### 4.2. Total RNA Extraction and Transcriptome Sequencing

After sampling, the samples were immediately frozen in liquid nitrogen and stored in a −80 °C ultra-low temperature refrigerator. Total RNA was extracted from the above samples according to the RNeasy plant mini kit (Qiagen, Hilden, Germany). The integrity of the RNA was tested using 1% agarose gel electrophoresis, and the quality and concentration of the RNA were quantitatively assessed using an Agilent Bioanalyzer 2100 system biochip analysis detector and a Nanodrop ND-1000 UV-Vis Spectrophotometer (Nanodrop Technologies, Wilmington, DE, USA). The qualified RNA samples were sent to PANOMIX Biomedical Tech Co., Ltd., Suzhou, China for cDNA library construction, and then high-throughput sequencing was performed using an Illumina HiSeqTM2500 sequencer (Illumina, San Diego, CA, USA) to generate paired-end sequences.

### 4.3. RNA-Seq and the Annotation

After RNA quality assessment, the maximum RIN value is 10, the minimum is 7.4, and the average value is 8.81. The results are acceptable. This project used Oligo (dT) magnetic beads to enrich mRNA with polyA structure and to fragment RNA into about 300 bp fragments. The first strand of cDNA was synthesized using RNA as a template, using a 6-base random primer and reverse transcriptase, and then the second strand of cDNA was synthesized using the first strand of cDNA as a template. After library construction, library fragment enrichment was performed using PCR amplification, and then library selection based on fragment size was about 450 bp, which was the total length of the RNA, adapter, and primer. Subsequently, the libraries were inspected using an Agilent 2100 Bioanalyzer (Agilent Technologies, Santa Clara, CA, USA), and the total and effective concentrations were tested. Libraries containing different index sequences were mixed in a scale based on the effective concentration and the amount of data needed. The mixed libraries were uniformly diluted to 2 nM and then formed into single-stranded libraries by base denaturation. After RNA extraction, purification, and library building, the samples were processed (Next-Generation Sequencing, NGS) with paired-end (PE) sequencing based on the Illumina sequencing platform.

### 4.4. Transcriptome Data Analysis

Firstly, filter the raw data using Fastp software version 0.20.0 [67] and then use Hisat2 version 2.2.1 [68] to align the high-quality sequences (Clean Data)—biological replicates for RNA-Seq and metabolomics obtained after filtering to the reference genome assembled by our research group. Based on the alignment results, use FeatureCounts version 2.0.1 [69] to calculate the expression level of each gene. On this basis, use DeSeq2 software version 1.36.0 to analyze the expression differences of samples, use the KAAS tool for KEGG annotation, and use the Pheatmap software version 1.0.12 package in R language to perform two-way clustering analysis.

### 4.5. Metabolite Extraction and Detection

Metabolites are the final expression products of an organism subject to genetic control and environmental influences. The above 9 avocado samples were sent to PANOMIX Biomedical Tech Co., Ltd., for qualitative and quantitative metabolite analysis using extensive targeted metabolomics techniques. The leaves were ground and dissolved in an extract, filtered through a microporous membrane, and metabolites were detected using liquid chromatography–tandem mass spectrometry (UPLC-MS/MS). Qualitative analysis of metabolites was carried out based on the information of UPLC-MS/MS secondary spectra, and the mass spectrum data of metabolites were detected using the multi-reaction monitoring of triple quadrupole mass spectrometry.

### 4.6. Metabolomics Data Analysis

Metabolomics data analysis mainly included the screening of differential metabolites and analysis of metabolic pathways. Metabolite mass spectrometry data were analyzed using Analyst 1.6.3 and Mutiaquant software version 3.0.1 with reference to the company’s self-built metabolite database. Through principal component analysis (PCA) of the samples, the overall metabolic differences between the samples and the variability of the samples within the group were obtained, so as to screen out differential metabolites. The metabolite content was normalized (total area normalization) using the range method, and the accumulation pattern of metabolites among different samples was clustered using R software version 4.3.2 (hierarchical cluster analysis, HCA). Finally, the KEGG database annotated the differential metabolites and classified their metabolic pathways.

Through the comprehensive application of these methods, we were able to comprehensively analyze the response mechanism of avocado to heat stress and to provide important reference and guidance for future studies.

## 5. Conclusions

In this study, we combined transcriptomic and metabolomic methods to investigate the response mechanisms of heat stress in avocado. The results showed that there was no significant change in leaf phenotype under short-term heat stress. Metabolomic analysis indicates that the metabolic pathways of ABC transporters, starch and sucrose metabolism, and phenylpropanoid biosynthesis are weakened under heat stress. The DEGs screened using transcriptome sequencing mainly involved plant hormone signal transduction, plant–pathogen interaction, and protein processing in endoplasmic reticulum et al. It is noteworthy to observe that, following two days of intense heat stress, avocado might mitigate the transportation of both Trehalose and Sinapoyl aldehyde through the modulation of the expression of *Hass.g03.10206* and *Hass.g03.10205* transport genes in its samples. This phenomenon indicates that the down-regulation of *Hass.g03.10206* and *Hass.g03.10205* genes, which are analogous to heat shock proteins, could potentially contribute to a reduction in the levels of Trehalose and Sinapoyl aldehyde. Conversely, an up-regulation of these genes may aid avocado in adapting to the challenging high-temperature environment. The results of this study will help to further understand the response mechanism to heat stress in avocado and other plants.

## Figures and Tables

**Table 1:** H0 vs. H1, changes in the common genes of the common metabolite Trehalose and Sinapoyl aldehyde under the H1 vs. H2 comparison group.

Gene_Id	H0 vs. H1	H1 vs. H2	NR	Pathway
*Hass.g03.10206*	up	down	class I heat shock-like protein	Protein processing in endoplasmic reticulum
*Hass.g03.10205*	up	down	class I heat shock-like protein	Protein processing in endoplasmic reticulum
*Hass.g02.08726*	down	up	8-hydroxylase-like protein	
*Hass.g04.14976*	up	down	putative aquaporin PIP2-8	

## Data Availability

The data generated or analyzed in this study are included in this article and its additional materials. The RNA-seq data reported in this paper have been deposited in the Genome Sequence Archive in the National Genomics Data Center (GSA: CRA017611) and are publicly accessible at https://ngdc.cncb.ac.cn/gsa, accessed on 18 September 2024.

## Supplementary Materials

The supporting information can be downloaded at: https://www.mdpi.com/article/10.3390/ijms251910312/s1.
